# Tyrosine kinase inhibitors for wet age-related macular degeneration: The current developmental landscape

**DOI:** 10.1016/j.jpet.2026.103803

**Published:** 2026-01-09

**Authors:** Rudra Amin, Peter K. Kaiser

**Affiliations:** 1 Drexel University College of Medicine, Philadelphia, Pennsylvania; 2 Cole Eye Institute, Cleveland Clinic, Cleveland, Ohio

**Keywords:** Wet age-related macular degeneration, Tyrosine kinase inhibitors, Vascular endothelial growth factor, Retina, Ophthalmology

## Abstract

Age-related macular degeneration (AMD) is a leading cause of permanent vision loss in older patients worldwide. The neovascular (wet) AMD is characterized by abnormal choroidal neovascularization driven by vascular endothelial growth factor (VEGF), platelet-derived growth factor, and Tie2 signaling pathways, leading to retinal damage and progressive vision decline. Current standard-of-care anti-VEGF therapies aim to limit choroidal neovascularization through extracellular targeting of cytokines involved in the VEGF signaling pathway implicated in angiogenesis. Although these existing therapies can be effective, many patients face a high treatment burden of multiple intraocular injections, which can negatively impact compliance, safety, and long-term efficacy. Tyrosine kinase inhibitors (TKIs) aim to address these limitations by offering longer durability, broad-spectrum targeting of angiogenic pathways, and a reduction in treatment burden through intracellular targeting of angiogenic pathways. With multiple pharmaceutical TKI candidates advancing through clinical trials and showing promising data, this class of drugs could lead to a shift in future treatment options for patients with wet AMD. Despite the progress TKIs have made, there have yet to be any candidates approved for wet AMD treatment. Much of the existing evidence is from early-phase and short-term studies, and questions remain about long-term efficacy and safety compared to current standard-of-care anti-VEGF therapies. Nevertheless, with multiple candidates advancing through phase III clinical trials, TKIs have the potential to emerge as a next-generation treatment class that may transform the wet AMD therapeutic landscape.

**Significance Statement:**

Given the chronic nature of wet age-related macular degeneration and the limitations of current anti-vascular endothelial growth factor therapies, tyrosine kinase inhibitors have emerged as a promising class of anti-angiogenic agents. This review highlights the recent clinical developments in this evolving therapeutic landscape.

## 1 Introduction

Age-related macular degeneration (AMD) is one of the leading causes of visual impairment that impacts over 18 million people 40 years and older in the United States, with around 1.5 million suffering from the late stages of AMD.^1^ AMD is among the most common causes of blindness, and approaches have evolved significantly to treat the disease before patients experience permanent vision loss.^2^ Early interventions involved photocoagulating abnormal retinal vessels; however, this procedure carried a high risk of long-term visual adverse effects. Currently, first-line treatment for wet AMD involves anti-vascular endothelial growth factor (VEGF) antibodies, which target one of the primary angiogenic pathways implicated in the progression of wet AMD. Corticosteroids may also be used to limit inflammation and vascular permeability; however, they increase the risk of cataract formation and intraocular pressure elevation. Emerging therapies include receptor tyrosine kinase inhibitors (TKIs), angiopoietin-Tie2 pathway inhibitors, and integrin pathway inhibitors.^3^

AMD is primarily driven by the aging of retinal pigment epithelium cells, which triggers a downstream cascade that results in the degeneration of the macular region of the retina.^4^ One such effect of this cascade is the increased production of VEGF because of insufficient blood supply and hypoxia.^5^ This may result in the formation of choroidal neovascularization (CNV), which, if left untreated, can lead to permanent vision loss.^5^ CNV is a hallmark of wet AMD, causing characteristic retinal edema, hemorrhage, and fibrosis, which leads to worse outcomes compared to dry AMD.^6^ Other signaling pathways, such as the platelet-derived growth factor (PDGF) and Tie2 pathways, are also implicated in CNV and the pathogenesis of wet AMD. The PDGF pathway facilitates interactions between endothelial cells and pericytes, which supports CNV.^7^ The Tie2 pathway is involved with stabilization of the vasculature and is inhibited with activation of the VEGF pathway, which results in abnormal vascularization and increased vascular permeability.^8^ Pairing Tie2 agonists with VEGF inhibition has been a proposed therapeutic combination that can be used to improve outcomes.^8^

Anti-VEGF therapies aim to limit the progression of CNV and reduce vision loss from the disease.^6^ Currently, approved anti-VEGF antibodies target the VEGF cytokines in the extracellular space, preventing receptor activation. Aflibercept is currently the most widely used anti-VEGF antibody to treat wet AMD, with faricimab, ranibizumab, bevacizumab, and brolucizumab also being used in clinical practice.^9^ Although these treatments are effective, they have less potency and a short half-life, resulting in the need for frequent administration of treatments, which can increase the risk of complications, such as endophthalmitis, and make patient compliance difficult.^10^ Additionally, current anti-VEGF therapies do not target other angiogenic pathways implicated in CNV, including the PDGF and Tie2 pathways. Combination therapies targeting multiple angiogenic pathways have shown promise in producing stronger results in patients with wet AMD; however, these therapies likely require a high treatment burden.^11^ TKIs are currently being developed to target the VEGF pathway and address some of these limitations to make treatment more accessible for patients with wet AMD.

Tyrosine kinases are enzymes that are involved in cellular signal transduction to modulate the metabolic activity of the cell through the phosphorylation of tyrosine residues. There are multiple subsets of tyrosine kinases, including receptor tyrosine kinases (RTKs), non-RTKs, and dual-specificity kinases. RTKs function through both an extracellular domain, which binds a ligand specific to the receptor, and an intracellular domain, which phosphorylates proteins to initiate a signal transduction cascade.^12^ The VEGF receptor (VEGFR), PDGF receptor (PDGFR), and Tie2 are RTKs that have been implicated in the pathogenesis of wet AMD because of their role in promoting angiogenesis and CNV.^13^ Non-RTKs are proteins that initiate signal transduction cascades in the cytoplasm.^14^ Dual-specificity kinases are tyrosine kinases that phosphorylate both tyrosine and serine/threonine residues on proteins.^15^ Because of the involvement of tyrosine kinases in multiple cellular pathways, there are multiple indications for TKIs, including various cancers, AMD, and idiopathic pulmonary fibrosis. Many TKIs that were originally developed as a therapeutic for cancer are now being tested in patients with wet AMD using different routes of administration to best target the retina.^16^ These TKIs can cause severe adverse effects when used systemically, so they have been repurposed for more targeted delivery for more localized diseases, such as wet AMD.

TKIs are a class of medications that block the function of tyrosine kinases and, therefore, inhibit the signaling pathway that the tyrosine kinase activates. Five binding modes of TKIs currently exist: type I inhibitors competitively bind the ATP-binding site of activated tyrosine kinases; type II bind the ATP-binding site of inactive tyrosine kinases; type III bind the allosteric pocket adjacent to the ATP site; type IV bind a distal allosteric site; and type V covalently bind to the ATP site.^17^^,^^18^ Type I–IV inhibitors are first-generation TKIs, whereas type V inhibitors are second-generation TKIs because they are irreversible inhibitors.^19^

**Fig. 1:**
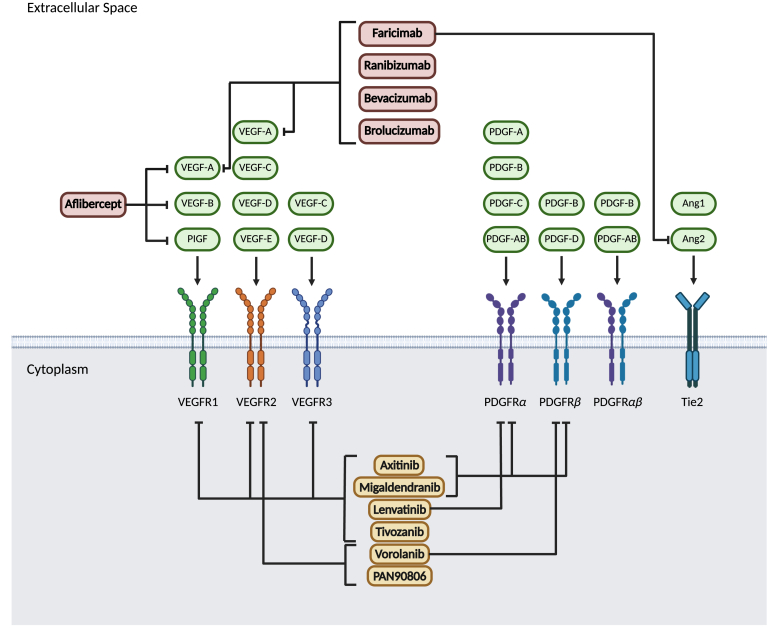
Key angiogenic targets involved in wet AMD pathogenesis and points of inhibition by approved anti-VEGF therapies and investigational tyrosine kinase inhibitors. Ang, angiogenin; PlGF, placental growth factor.

Using TKIs to block the downstream effects of VEGF receptor activation in wet AMD confers multiple advantages over current anti-VEGF compounds. One advantage is that TKIs act intracellularly at the kinase domain of the RTK and do not depend on the amount of VEGF in the extracellular space, whereas anti-VEGF antibodies target the VEGF pathway upstream by sequestering VEGF extracellularly before it can bind and activate VEGFR.^20^ Additionally, TKIs are not limited to VEGF pathways and can act across multiple other signaling pathways to inhibit signals for angiogenesis, whereas anti-VEGF antibodies generally target only VEGF-A and VEGF-C, which activate VEGFR1 and VEGFR2 (Fig. 1).^20^ This allows for a more complete inhibition of proangiogenic pathways regardless of VEGF abundance or isoform. Preclinically, anti-VEGF TKIs have been shown to inhibit VEGF-induced neovascularization up to 95% and VEGF-induced vascular permeability up to 100% in rat corneal micropocket models.^21^ TKIs have also demonstrated greater efficacy in inhibiting angiogenesis in vivo relative to the anti-VEGF antibody bevacizumab.^22^ Another advantage of using TKIs to treat wet AMD is that the small molecule nature of TKIs allows for use in sustained release systems that can decrease patient burden and reduce the risk of intraocular inflammation.^16^ This review evaluates an emerging class of TKIs in development for wet AMD, highlighting their mechanisms of action, current clinical evidence, and potential to overcome limitations of existing treatment options and improve patient outcomes.

## 2 Current therapies in development

### 2.1 Axitinib

Axitinib is a second-generation TKI that potently targets the VEGFR1, VEGFR2, and VEGFR3 tyrosine kinase receptors.^23^ It also inhibits the PDGFR*α* and PDGFR*β* tyrosine kinases and c-Kit receptors at higher concentrations.^23^ Among TKIs currently under investigation in ophthalmology, axitinib has the highest potency for the VEGF receptors.^24^ Several axitinib-based therapies are in development for wet AMD and other retinal diseases, including Axpaxli (also known as Ocular Therapeutix-TKI, Ocular Therapeutix), CLS-AX (Clearside Biomedical), AR-14034 (Alcon), and GLK-401 (Glaukos).

Among the axitinib-based formulations in development, Axpaxli is the furthest advanced with phase III clinical results expected in 1Q2026. Axpaxli is an investigational intravitreal, hydrogel implant that delivers axitinib with 0-order kinetics for 6–12 months, reducing the need for frequent intravitreal injections. In a phase I trial, where 21 patients with anti-VEGF treatment-experienced wet AMD received either an Axpaxli 600 *μ*g implant on day 0 with aflibercept 2 mg at month 1 or aflibercept 2 mg every 8 weeks, it was found that the treatment was well tolerated with no drug-related adverse effects reported in the Axpaxli arm. At 7 months, best-corrected visual acuity (BCVA) and central subfield thickness (CST) remained stable in both groups.^25^ Among the patients who received Axpaxli in this trial, 80% remained rescue-free at week 24 and 60% at week 52.^26^ For those patients in the treatment arm of the study who received a supplemental anti-VEGF injection, the median time to injection was 44.6 weeks.^26^ Another phase I trial studying dose escalation of Axpaxli in 29 patients with wet AMD (both treatment-naïve and treatment-experienced included) found it to be well tolerated at the highest dose administered (3 × 200 *μ*g implants).^26^ Axpaxli is currently being investigated in phase III clinical trials. SOL-R is a noninferiority study comparing repeat Axpaxli injections every 6 months to repeat 2 mg aflibercept injections every 8 weeks to evaluate the mean change in BCVA from baseline at 56 weeks in patients with wet AMD.^27^ The phase III SOL-1 clinical trial is comparing a single Axpaxli injection to a single 2 mg aflibercept injection in patients with treatment-naïve wet AMD to determine the superiority and durability of Axpaxli at 36 weeks.^28^ Both studies are fully enrolled.^29^

Axitinib is also being evaluated for the treatment of wet AMD through a suprachoroidal injection as CLS-AX. Suprachoroidal delivery provides the advantage of targeting the posterior segment, including the retina, of the eye more directly compared to intravitreal administration.^30^ A phase I/IIa trial (OASIS) studied a dose escalation of CLS-AX from 0.03 to 1 mg of axitinib in 27 patients with treatment-experienced wet AMD. This trial demonstrated that CLS-AX is well tolerated with no reports of serious adverse effects at 3 months posttreatment.^31^ Patients treated with 0.5 or 1 mg had a 73%–100% reduction in treatment burden after 3 months, whereas the BCVA and CST of patients in the trial remained stable throughout the 3 months of study.^31^ In those who did not respond as well to previous anti-VEGF infections, optical coherence tomography (OCT) showed anatomical signs of TKI activity.^31^ An extension of the OASIS study followed 14 patients with treatment-experienced wet AMD receiving either 0.1, 0.5, or 1 mg for 3 additional months beyond the original phase I/IIa trial. Similar findings were found at 6 months posttreatment in these groups, with 67% of patients in the trial remaining injection-free through 6 months.^32^ A phase IIb trial (ODYSSEY) analyzed changes in BCVA, safety, and tolerability to 36 weeks in 60 patients with treatment-experienced wet AMD randomized to receive CLS-AX or aflibercept. Unlike the OASIS trial, this trial involved patients who received multiple doses of CLS-AX, with 2 (5%) patients receiving 1 dose, 32 (80%) patients receiving 2 doses, and 6 (15%) patients receiving 3 doses across the 36 weeks of the trial.^33^ Across the 36 weeks, most patients receiving CLS-AX experienced decreased treatment burden, with 67% remaining rescue injection-free through week 24, and an 84% reduction in injection frequency.^33^ Visual and anatomical outcomes were stable, with mean BCVA maintained within 2 letters at 24 and 36 weeks, and CST fluctuations reduced compared to the aflibercept arm.^33^ CLS-AX was generally well tolerated with 4 cases of intraocular inflammation that resolved by week 36 of the study and no treatment-related severe adverse events.^33^ Because of the positive results from the OASIS and ODYSSEY trials, a phase III trial is currently being planned to evaluate noninferiority to every 8-week aflibercept 2 mg and investigate a flexible dosing regimen between 3 and 6 months.^33^

Alcon is currently developing AR-14034 as an intravitreal implant for the sustained release of axitinib. Preclinical studies demonstrated a significant reduction in retinal vessel leakage when the product was implanted in rabbits and miniature swine.^34^ The 2-stage phase I/II NOVA-1 trial is currently recruiting patients to study both dose escalation and safety, preliminary treatment effects, and durability compared to aflibercept.^35^

Glaukos Corporation is currently developing an intravitreal implant containing axitinib (GLK-401) for both AMD and diabetic macular edema (DME).^36^ This candidate is currently being evaluated in phase II trials.^36^

### 2.2 Vorolanib

Vorolanib is a TKI that competitively binds to and selectively inhibits kinase insert domain receptor (VEGFR2), PDFGR*β*, FMS-like tyrosine kinase 3, and c-Kit.^37^ It is a sunitinib derivative that was designed with a shorter half-life to reduce the accumulation of the compound in tissues and greater selectivity through weaker inhibition of rearranged during transfection (RET) and AMP-activated protein kinase *α*1.^37^^,^^38^ Vorolanib also does not bind melanin, which reduces the risk for adverse effects related to drug accumulation in pigmented ocular tissues.^22^ Originally developed to treat advanced solid tumors, it is now being tested to treat AMD because of its antiangiogenic effects.

X-82 (Tyrogenex) was an oral vorolanib product initially developed for wet AMD; however, it was discontinued because of safety issues. Although the X-82 therapy in combination with as-needed anti-VEGF injections showed noninferiority of visual acuity compared to a placebo group, phase I and II trials reported multiple gastrointestinal and hepatobiliary adverse events.^39^^,^^40^ A phase I clinical trial testing X-82 in 35 patients with wet AMD (7 treatment-naïve) demonstrated reversible elevated liver enzymes after X-82 administration. The APEX phase II clinical trial with 157 patients with wet AMD was discontinued because of a limited benefit-to-risk profile.^39^^,^^40^

Duravyu (EyePoint Pharmaceuticals) is an intravitreal insert that continuously delivers vorolanib over a period of 6 months through EyePoint Durasert E technology. In the phase I DAVIO trial testing Duravyu in 17 patients with treatment-experienced wet AMD, the product was well tolerated with no reports of ocular or drug-related systemic severe adverse events.^41^ At 12 months, 35% of patients in the trial did not need a supplemental injection, with a 73% reduction in treatment burden.^41^ Visual and anatomic outcomes remained stable over 12 months, with a reduction in BCVA by 4.1 letters and CST by 2.8 *μ*m.^41^ The phase II DAVIO 2 trial enrolled 161 treatment-experienced patients, compared a 2-mg low dose of Duravyu, a 3-mg high dose of Duravyu, and a 2-mg dose of aflibercept. BCVA and CST of the Duravyu treatment arms remained stable throughout the 12 months of the study, with limited fluctuations in CST when compared to the aflibercept arm of the study.^42^ The DAVIO 2 study also found that at 14 months, 43% of patients in the low dose arm and 46% in the high dose arm remained free of supplemental injections, compared with 65% in the aflibercept arm.^42^ The Daravyu treatment arms also showed an 80%–81% reduction in treatment burden and were well tolerated with no treatment-related ocular or systemic severe adverse events.^42^ The phase III LUGANO and LUCIA trials for wet AMD are fully enrolled, with results expected in 2026. They are identical noninferiority trials comparing 2.7 mg Duravyu to 2 mg aflibercept, dosed every 8 weeks, and have a 1-year efficacy and safety endpoints.^42^^,^^43^

### 2.3 Lenvatinib

Lenvatinib is a TKI that targets VEGFR1-3, PDGFR*α*, fibroblast growth factor receptor 1-4, RET, and c-Kit.^44^ AIV007 (AiViva Biopharma) uses lenvatinib in a proprietary periocular gel formulation to treat wet AMD, benign prostatic hyperplasia, and solid tumors. AIV007 is currently in a phase I trial with 18 patients with treatment-experienced AMD or DME.^45^ The preliminary results of the phase I trial suggest a well tolerated safety profile, with only mild or moderate ocular adverse events reported.^46^ Additionally, AIV007 has demonstrated a 16-letter gain in BCVA at 84 days and an average of a 4-letter gain at 168.^46^ For 4 patients who have completed the trial, there has been a stable or improved CST through 168 days.^46^

### 2.4 Migaldendranib

Migaldendranib is being developed as D-4517.2 (Ashvattha Therapeutics) as a subcutaneously administered nanomedicine containing hydroxyl dendrimers attached to TKI molecules, with the goal of improving convenience for patients. Additionally, this drug can cross the blood–retinal barrier into the eye to selectively target VEGFR1-3, PDGFR*α*, PDGFR*β*, c-Kit, and colony-stimulating factor 1 receptor in retinal pigment epithelial cells and microglia in CNV lesions.^47^ Renal clearance of the drug minimizes systemic and hepatic toxicity.^47^ D-4517.2 was well tolerated up to the highest dose of 2 mg/kg in a phase I trial with 12 healthy patients.^48^ An ongoing phase II study with 50 patients with treatment-experienced wet AMD or DME is assessing chronic dosing of D-4517.2, with administration every 2 or 4 weeks for up to 40 weeks.^49^ Results from 8 patients with wet AMD who completed the trial demonstrated a 79.9% reduction in anti-VEGF treatment burden at 24 weeks after treatment compared to 24 weeks before screening.^50^ Additionally, the mean BCVA improved by 3 letters in the study eye, and the mean CST improved by 45.5 *μ*m at 24 weeks.^50^ No serious or ocular adverse effects were reported, and injection site reactions were reported in 3 patients with AMD and 2 patients with DME with a total of 26 injection site reactions among 302 injections across all patients in the study.^50^ An oral formulation is currently in preclinical development, with preliminary data showing that an oral dose of migaldendranib significantly reduces CNV lesion area and vascular leakage in laser-induced CNV mouse models and *VLDLR*^*-/-*^ mouse models.^48^

### 2.5 PAN90806

PAN90806 (Zhaoke Ophthalmology) is an anti-VEGFR2 TKI in development as an eye drop formulation with the aim of increasing patient compliance. In an initial phase I/II trial involving 40 patients with treatment-naïve wet AMD taking the eye drops daily for 8 weeks, where patients received 1 of 5 different doses escalating from 1 to 4 mg/mL, dose-dependent keratopathy was observed in those who received the 3 highest doses.^51^ The cohorts for the 3 highest doses of the study did not have full enrollment due to these findings and the lower 2 doses (1, 2 mg/mL) were used to analyze the efficacy of PAN90806.^51^ Those who received the 1 mg/mL dose had an average of a 12-letter increase in BCVA at 8 weeks, whereas those who received the 2 mg/mL dose lost an average of 1 letter in BCVA at 8 weeks.^51^ Additionally, both cohorts demonstrated a decrease in the average CST, center point thickness, total lesion size, and area of fluorescein leakage, with a greater effect in the 2 mg/mL cohort in the first 3 measurements.^51^ Because of the adverse events reported in this study, a new formulation was developed to limit corneal exposure. A phase I/II trial using this formulation was conducted with 51 treatment-naïve patients getting either a 2, 6, or 10 mg/mL dose of PAN-90806 once a day for 12 weeks.^52^ At least 1 treatment-related adverse event occurred in 17.6% of patients enrolled in this study, with 9.8% of patients reporting a total of 6 corneal adverse events related to the treatment.^52^ Overall, 51% of patients remained injection-free through the course of the study, and the mean number of rescue ranibizumab injections was 0.84 per patient, representing a 79.4% reduction in treatment burden.^53^ The study also found that there was a 79.4% decrease in treatment burden. PAN90806 is currently undergoing animal studies in preparation for further clinical trials.^54^

### 2.6 Tivozanib

Tivozanib is a TKI targeting VEGFR1, VEGFR2, and VEGFR3, currently approved to treat advanced renal cell carcinoma, and currently being developed for wet AMD and DME as KHK4951 (Kyowa Kirin) a nano-crystallized eye drop formulation.^55^ A phase I dose escalation study in Japan with 116 enrolled patients compared 3 cohorts: a single dose of tivozanib once in 40 healthy patients, 3 doses daily for 21 days in 48 healthy patients, and 3 doses daily for 21 days for 28 patients with treatment-experienced wet AMD.^56^ All cohorts had a group receiving a placebo dose and a group receiving a treatment dose.^56^ No serious adverse events were reported, and the most common adverse event in those receiving multiple doses was reversible punctate keratitis, occurring in 47.2% of the healthy cohort and 14.3% of the wet AMD cohort.^56^ In the wet AMD cohort, BCVA remained stable with no patient experiencing a decrease of more than 10 letters from baseline.^56^ The CST changes were −27.6 ± 54.88 *μ*m in patients with wet AMD receiving 1 drop of 0.5 w/v% of tivozanib, −35.6 ± 49.64 *μ*m in patients with wet AMD receiving 1 drop of 1.0 w/v% of tivozanib, and −43.7 ± 55.19 *μ*m in patients with wet AMD receiving 2 drops of 1.0 w/v% of tivozanib.^56^ OCT measurement confirmed significant anatomic improvements in many patients in the wet AMD cohort.^56^ A phase II study is currently recruiting patients to further test KHK4951 in patients with wet AMD.

## 3 Discussion

### 3.1 Current challenges and future directions

Despite the promise recent clinical trials have shown, several obstacles remain before TKIs can be considered an effective treatment option for patients with wet AMD. Most of the current data on the efficacy and safety of TKIs for wet AMD comes from early-phase or short-term trials; therefore, questions remain about the long-term safety and efficacy of these drugs (Table 1). This concern is especially relevant as TKIs often have longer intraocular half-lives and broad-spectrum target profiles compared to current anti-VEGF therapies. Given that wet AMD is a chronic disease, patients will likely need prolonged treatment with TKIs, highlighting the critical need to have data on their long-term effects. Additionally, there have been no large-scale, randomized clinical trials comparing TKIs to current gold standard anti-VEGF agents such as aflibercept or ranibizumab. Without these studies, the relative clinical value and cost-effectiveness of TKIs remain uncertain. Although ongoing and planned phase III trials will likely answer many of these questions, more targeted and long-term clinical trials will be needed to truly understand the safety and efficacy of TKIs in patients with wet AMD. Ultimately, closing these gaps in knowledge will be vital for advancing TKIs toward clinical adoption as a next-generation therapy for wet AMD.

**Table 1:** Key results from clinical trials testing TKIs in patients with wet AMD

Drug Name	Active TKI	Delivery	Visual Acuity Outcome (BCVA)	CST Outcome	Treatment Burden Reduction/Durability	Safety Profile	Current Clinical Trial Phase
Axpaxli	Axitinib	Intravitreal implant	Stable at 7 mos	Stable at 7 mo	60% rescue-free at wk 52; median 44.6 wk to rescue	Well tolerated; no drug-related SAEs	Phase III
CLS-AX	Axitinib	Suprachoroidal injection	Stable at 24 and 36 wk	Stable at 36 wk with reduced fluctuation	67% injection-free at 24 wk, 84% reduced injection frequency at 36 wk	4 transient intraocular inflammation cases; no SAEs	Phase III
AR-14034	Axitinib	Intravitreal implant	TBD	TBD	TBD	TBD	Phase I/II
GLK-401	Axitinib	Intravitreal implant	TBD	TBD	TBD	TBD	Phase II
Duravyu	Vorolanib	Intravitreal implant	Stable for 12 mo	Stable at 12 mo with reduced fluctuations	80%–81% treatment burden reduction	Well tolerated; no ocular or systemic SAEs	Phase III
AIV007	Lenvatinib	Periocular gel	16-letter gain at 84 d, 4-letter gain at 168 d	Stable or improved at 168 d	TBD	Mild-to-moderate ocular AEs only	Phase I
D-4517.2	Migaldendranib	Subcutaneous injection	3-letter gain at 24 wk	45.5 *μ*m reduction at 24 wk	79.9% treatment burden reduction at 24 wk	No ocular or serious AEs; mild injection site reactions	Phase II
PAN90806	anti-VEGFR2 TKI	Eye drop	12-letter gain with 1 mg/mL; 1-letter loss with 2 mg/mL (both at 8 wk)	Decrease at 8 wk	79.4% treatment burden reduction at 12 wk; 51% remained injection-free at 12 wk	Reformulated due to keratopathy in the initial phase I trial; 17.6% of patients had at least 1 treatment-related AE with 9.8% having a corneal AE with the reformulation	Phase I/II
KHK4951	Tivozanib	Eye drop	Stable at 21 d	Anatomical OCT improvements	TBD	Reversible punctate keratitis in some patients; no SAEs	Phase II

Abbreviations: AE, adverse event; OCT, optical coherence tomography; SAE, serious adverse event; TBD, to be determined.

## 4 Conclusions

TKIs are a promising new class of anti-VEGF therapies for wet AMD, with preliminary studies indicating treatment outcomes comparable to current anti-VEGF therapies paired with decreased treatment burden. Although there are currently no Food and Drug Administration-approved TKIs for AMD, our review highlights multiple candidates with promising results and desirable safety profiles, especially Axpaxli, CLS-AX, and Daravyu, which are all currently in phase III trials. Currently, most trials have been small and/or with short follow-ups, leaving critical questions about long-term safety and durability unanswered. Larger trials with more variability in dosing regimens and longer follow-up times should provide greater insight into how this class of drugs could impact future clinical treatment of patients with wet AMD. In conclusion, TKIs for wet AMD are an evolving and rapidly changing landscape that has the potential to reshape clinical management of wet AMD.

## Conflict of interest

Peter K. Kaiser reports a relationship with AAVAntgarde Bio, including board membership and equity or stocks. Peter K Kaiser also reports consulting or advisory. relationships with AbbVie Inc, Alexion, Alkeus Pharmaceuticals Inc, Allgenesis, Alzheon Inc, Annexon Biosciences, Asclepix Therapeutics Inc, Astellas Pharma Inc, Augen Therapeutics, Aviceda, Bayer Corporation, Bausch and Lomb, Beacon Therapeutics, Biogen Inc, Carl Zeiss Meditec Inc, Celltrion Healthcare Co, Ltd, Complement Therapeutics, Endogena Therapeutics Inc, Frontera Therapeutics Inc, Galimedix, Innovent Biologics Inc, Invirsa, Isarna Therapeutics GmbH, Janssen Pharmaceuticals Inc, jCyte, KANAPH Therapeutics Inc, Chengdu Kanghong Pharmaceuticals Group Co Ltd, KERA, Kriya Therapeutics Inc, Santec Holdings Corporation, Nanoscope, Ocugenix, Omeros Corporation, Osanni Bio, Panther Pharmaceuticals, Ray Therapeutics, REGENXBIO Inc, Resonance Medicine Inc, Restore Vision, Retinal Sciences, ReVana, Roivant, Samsung Bioepis Co, Ltd, Sandoz Inc, SGN Nanopharma, Smile Biotek Zhuhai Ltd, Stealth BioTherapeutics Inc, Stuart, Sudo Biosciences Inc, Sustained Nano Systems, Thea, Tilak, Unity Biotechnology Inc, Vanotech, VisgenX Peter K. Kaiser reports a relationship with Amaros, iRenix, Oculis, RevOpsis Therapeutics Inc, including consulting or advisory and equity or stocks. Peter K. Kaiser also reports a relationship with Ocular Therapeutix Inc, including employment and equity or stocks. All other authors declare no conflicts of interest.
